# Food consumption, physical activity and aerobic capacity in systemic lupus erythematosus patients with high cardiovascular risk

**DOI:** 10.1016/j.clinsp.2024.100418

**Published:** 2024-07-13

**Authors:** Fabiana Infante Smaira, Bruna Caruso Mazzolani, Sofia Mendes Sieczkowska, Marina Romero, Thainá Toledo Ribeiro, Milla Cordeiro Amarante, Sandra Pasoto, Ana Lúcia de Sá Pinto, Fernanda Rodrigues Lima, Fabiana Braga Benatti, Hamilton Roschel, Bruno Gualano

**Affiliations:** a Applied Physiology and Nutrition Research Group – Center of Lifestyle Medicine; Laboratory of Assessment and Conditioning in Rheumatology, Hospital das Clínicas, Faculdade de Medicina, Universidade de São Paulo (HCFMUSP), São Paulo, SP, Brazil; b Rheumatology Division, Faculdade de Medicina, Universidade de São Paulo (FMUSP), São Paulo, SP, Brazil; c Faculdade de Ciências Aplicadas, Universidade Estadual de Campinas (UNICAMP), Campinas, SP, Brazil

**Keywords:** Systemic lupus erythematosus, Functional capacity, Aerobic capacity

## Abstract

Systemic Lupus Erythematosus (SLE) is a chronic, autoimmune and multisystemic rheumatic disease. Patients with SLE have decreased functional and aerobic capacity, as well as increased prevalence of Cardiovascular Diseases (CVD), which are the primary causes of morbimortality in this condition. Dietary intake and physical activity are well-known modifiable cardiovascular risk factors. The aim of this study is to describe food consumption, sedentary behavior, physical activity level, and functional and aerobic capacity in a sample of SLE patients with high cardiovascular risk. This was a cross-sectional study in which patients were assessed for (i) Demographic, anthropometric, and disease-related parameters; (ii) Food consumption; (iii) Physical activity level and sedentary behavior; (iv) Functional and aerobic capacity. Patients averaged 41.7 ± 9 years, and most were classified as overweight/obese (87%). Average macronutrient intake was within recommendations; however, fiber (16 ± 9g) and calcium (391 ± 217 mg) intakes were below, and sodium intake (2.9 ± 1.3 mg) was above recommendations. Besides, food consumption assessed by the Nova system showed a predominance of unprocessed foods (43.8 ± 14.0%TEI), although ultraprocessed food intake (20.0 ± 13.9%TEI) was slightly higher than that seen in the Brazilian population. Patients also exhibited high sedentary behavior (8.2 ± 2.2h) and only eighteen participants reached the minimum recommended amount of moderate-to-vigorous physical activity. Overall, patients had a low functional and aerobic capacity compared to the general population. Data from this study may help design dedicated clinical trials aiming to investigate the effects of lifestyle intervention to mitigate CVD in SLE.

## Highlights

• Patients with systemic lupus erythematosus have decreased functional and aerobic capacity and increased prevalence of cardiovascular diseases.
• An unhealthy diet, characterized by a high consumption of ultraprocessed foods, as well as physical inactivity increase the cardiovascular risk factor.
• 58.6% of the patients exhibited high sedentary behavior (above 8h/day) and none of them meet with minimum physical activity recommendation.
• Our sample reported a slightly higher consumption of ultraprocessed foods than that of the general population (20.0% vs. 18.4% of total energy value).

## Introduction

Systemic Lupus Erythematosus (SLE) is a chronic, autoimmune, and multisystemic rheumatic disease characterized by the production of autoantibodies that target the body's own tissues and organs, leading to inflammation and damage.^1^ SLE is more prevalent in women, and its symptoms can vary widely, ranging from mild joint pain and rashes to severe organ damage, among other complications. ^2^

Individuals with SLE are at an increased risk of developing Cardiovascular Diseases (CVD), the leading cause of morbidity and mortality in this population. ^3^ Several factors contribute to the increased CVD risk in SLE, including chronic inflammation, endothelial dysfunction, accelerated atherosclerosis, and traditional cardiovascular risk factors such as hypertension, diabetes, dyslipidemia, and obesity.^3^ Importantly, other lifestyle factors such as poor diet, sedentary behavior and physical inactivity may also contribute to the increased CVD risk in these patients.^4^^,^^5^

Food consumption is a well-known modifiable cardiovascular risk factor. A diet rich in unprocessed foods is associated with better cardiovascular health parameters (e.g., body mass index, blood pressure, lipid, and glucose profile).^6^^,^^7^ Nonetheless, an unhealthy diet, characterized by high consumption of ultra-processed foods – those generally rich in calories, sugar, fat, and salt and poor in fibers, protein, vitamins, and minerals – has been associated with hyperglycemia, hyperinsulinemia, dyslipidemia, hypertension, and obesity.^8^^,^^9^ Moreover, in SLE, specific dietary components, such as omega-3 fatty acids, antioxidants, and fiber, have been shown to have anti-inflammatory and cardio-protective effects.^10^ However, studies characterizing food consumption in SLE patients remain scarce, so it is difficult to conclude the actual impact of food intake in the patient's cardiovascular health.

Even on remission, patients with SLE often show physical activity levels below the recommended guidelines^11^ as well as greater time spent in sedentary behavior when compared with the general population. ^12^^,^^13^ These behaviors, combined and independently, are important risk factors for cardiovascular diseases and mortality and decreased functional and aerobic capacity.^14^^,^^15^ Regular exercise, ranging from moderate to vigorous intensity, is widely acknowledged as a crucial element in the prevention and management of chronic illnesses.^16^ In SLE, exercise has been found to reduce inflammatory markers, mitigate cardiometabolic risk factors, and improve functional and aerobic capacity.^14^^,^^17^

Given the high prevalence and impact of CVD in SLE, identifying modifiable risk factors in this population is of utmost importance. Therefore, the aim of the study was to describe food consumption, sedentary behavior, physical activity level, and aerobic and functional capacity in a sample of SLE patients with high cardiovascular risk.

## Methods

### Study design and participants

This cross-sectional study was conducted at the Clinical Hospital, School of Medicine, University of São Paulo using data from participants who were screened for participation in a clinical trial of a lifestyle intervention vs. control (clinicaltrials.gov, NCT04431167), carried out between August 2020 and March 2023 in São Paulo, Brazil.

The study sample comprised patients diagnosed with SLE from our outpatient clinic at the university hospital. Inclusion criteria were as follows:(1) Female; (2) Age between 18 and 65 years; (3) SLEDAI score ≤ 4 (disease in remission); (4) Having high cardiovascular risk (i.e., Body Mass Index [BMI] between 25 and 40 kg/m^2^ and/or dyslipidemia, hypertension, or diabetes). Patients who were illiterate or had cognitive impairments that could compromise data collection were excluded. The study was approved by the local Ethical Committee (Commission for Analysis of Research Projects, CAPPesq; approval: 19554719.5.0000.0068), and all patients provided informed consent before participating. The procedures were conducted in accordance with the Declaration of Helsinki revised in 2008.

Patients were assessed for: (i) Demographic (i.e., age, income, educational and marital status), anthropometric (i.e., height and weight), and disease-related (i.e., disease duration, disease activity and pharmacological treatment) parameters; (ii) Food consumption; (iii) Physical activity level and sedentary behavior; (iv) Functional capacity and (v) Aerobic capacity.

### Demographic, anthropometric and disease-related parameters

Demographic and disease-related parameters were obtained through patients' medical records and interviews. Global health status and pain were evaluated by means of the Visual Analogue Scale (VAS), a 10-point grading scale.^18^ Height was measured with a stadiometer, weight with a calibrated scale, and waist circumference with plastic tape at the midpoint between the last floating vertebra and the iliac crest.

### Food consumption

Food consumption data was collected using 24-hour food recalls on three nonconsecutive days, including one weekend day, and analyzed using the Dietbox software online version. Standardized recipes were used to quantify foods and ingredients for preparations such as soups, purees, pies, and sandwiches. To characterize participants' dietary intake, the authors calculated total energy intake in kilocalories and macronutrients in grams and as a percentage of Total Energy Intake (%TEI)^19^ as well as energy contribution (%TEI) per food processing level according to previous criteria.^20^

The Nova system categorizes foods and food products based on their processing level and purpose. It includes four main groups: (1) Unprocessed or Minimally Processed (UNMP) foods, which are natural foods altered through processes that preserve them, making them suitable for storage, safe, and pleasant to consume (e.g., dried fruits, seeds, milk); (2) Processed Culinary Ingredients (PCI), derived from nature or Group 1 foods through processes like pressing, refining, and drying (e.g., salt, sugar, oils); (3) Processed foods (PR), essentially made by adding salt, oil, sugar, or other substances from Groups 1 and 2 (e.g., homemade bread, cheese, dried meat); (4) Ultra-processed foods (UPR), mainly made from food-derived substances and additives, with little or no intact food from Group 1 (e.g., snacks, sausages, candies).^20^

### Physical activity level and sedentary behavior

Physical activity level and sedentary behavior were objectively measured using a triaxial ActivPAL® accelerometer (PAL Technology), which allowed the evaluation of the time spent in sedentary behavior, light, and moderate-to-vigorous intensity physical activities. All patients were instructed to wear the accelerometer for 7 days, removing it only during submerged water activities (swimming pool). The accelerometer was positioned on the medial portion of the right thigh using a waterproof bandage. Patients had to accumulate at least 10 hours of valid activity per day for at least 4 days. Additionally, patients had to fill in a device use diary, which included the day and time of wear and removal of the device. The collected data was downloaded to the computer through the ActivPAL® software. The following data were reported: (1) Sitting time (hours/day); (2) Light-intensity physical activity (minutes/day), (3) Moderate-to-vigorous physical activity (minutes/day and minutes/week); (4) Number of steps.

### Functional capacity

The Timed-Stands Test consisted of counting the number of times the patient could stand up and sit down from a chair using only the lower limbs for 30 seconds.^21^ The Timed Up-and-Go test assessed the time required for the patient to stand up from a chair, walk 3 meters, turn 180 degrees, return and sit on the chair.^22^ Patients had 2 attempts in each test and the average between attempts was considered for analysis. All patients underwent a familiarization session, held at least 48 hours before the actual test, to ensure data reliability.

Handgrip strength was evaluated using a hand dynamometer. The test was performed with participants using their dominant hand and remaining in an upright position with the arm extended along the body. Participants perform maximum handgrip strength on the device for 5 seconds. The average value of 3 attempts, separated by a 1-minute interval, was considered for analysis.^23^

### Aerobic capacity

During the cardiopulmonary exercise test, cardiovascular behavior was continuously evaluated using an electrocardiograph with 12 simultaneous leads. Heart rate was recorded throughout the test and blood pressure was recorded at rest and at the end of the effort. Maximal aerobic capacity was assessed through the direct measurement of oxygen consumption at peak exercise (VO_2_peak) using a breath-by-breath sensor system (Metalyzer model III b/breath-by-breath). VO_2_peak was considered as the average value in the last 30 seconds of effort.^24^ The test was considered maximal when two of the following four criteria were met: (1) Incidence of a plateau in VO_2_; (2) Respiratory exchange ratio above 1.10; (3) Heart rate greater than 90% of the maximum predicted for age; (4) Perceived exertion ≥ 17. Metabolic thresholds were determined by a single experienced evaluator based on the responses of the ventilatory equivalents of O_2_ (VE/VO_2_) and CO_2_ (VE/VCO_2_). The ventilatory anaerobic threshold was considered as the point where there was an abrupt increase in VE/VO_2_ without a concomitant increase in VE/VCO_2_. The respiratory compensation point was considered as the moment in which ventilatory equivalents showed a similar increase.^25^ All tests were conducted under the supervision of a physician. Before the exercise test, participants were instructed not to drink caffeinated beverages and not to engage in vigorous physical activity in the 24 hours prior to the exam.

### Statistical analysis

Descriptive analyses were performed using R (version 4.2.2, R Core Team, Vienna, Austria). All data are expressed as mean ± standard deviation or frequency. The Raincloud Plots were generated using the following packages: “ggplot2” for data visualization and “ggdist” for probability distribution plots.

## Results

A total of 99 women (age: 41 ± 9 years) diagnosed with SLE were included in the study. Most patients reported being employed (64.6%), in a stable relationship (55.6%), with a complete high school or incomplete academic degree (55.5%), from socioeconomic class D/E (e.g., until US$ 565.3) (59.4%), and classified as overweight/obese (87.0%). Average waist and hip circumferences were 95.6 ± 10.7 cm and 109.0 ± 10.0 cm, respectively. Additionally, they reported moderate global health (5.0 ± 2.9) and pain (4.6 ± 3.0), and the main pharmaceutical treatments included Disease-Modifying Anti-Rheumatic Drugs (DMARDs), immunosuppressants, and antihypertensives (Table 1).

**Table 1:** Demographic, anthropometric and disease-related parameters in systemic lupus erythematosus patients with high cardiovascular risk profile.

	M ± SD or n (%)
**Age (years) (*n* = 99)**	41 ± 9
**BMI (kg/m²) (*n* = 99)**	29.5 ± 4.4
*BMI Classification*	
Eutrophic	13 (13.1)
Overweight	44 (44.4)
With obesity	42 (42.4)
**Waist circumference (cm) (*n* = 99)**	95.6 ± 10.7
**Hip circumference (cm) (*n* = 99)**	109.0 ± 10.0
**Income (*n* = 99)**	
Class A	0 (0)
Class B	3 (3.1)
Class C	36 (37.5)
Class D/E	57 (59.4)
**Working (Yes) (*n* = 99)**	64 (64.6)
**Smoking (Yes) (*n* = 99)**	11 (11.1)
**Practice physical exercise (Yes) (*n* = 99)**	5 (5.1)
**Stable relationship (*n* = 99)**	55 (55.6)
**Educational level (*n* = 99)**	
Incomplete elementary school	6 (6.1)
Elementary school/ Incomplete high school	16 (16.2)
High school/ Incomplete academic degree	55 (55.5)
Complete academic degree	22 (22.2)
**Disease parameters (*n* = 99*)***	
Duration (years)	14 ± 12
SLEDAI	1.0 ± 1.9
SLICC	0.6 ± 1.1
VAS global health (0-10)	5.0 ± 2.9
**Pharmacological treatment (*n* = 99)**	
Biological drugs	19 (19.2)
DMARDs	77 (77.8)
NSAIDs	5 (5.1)
Glucocorticoids	46 (46.5)
Immunosuppressants	55 (55.6)
Muscle Relaxant	16 (16.2)
Painkillers	22 (22.2)
Antihypertensives	57 (57.6)
Statins	20 (20.2)
Anticoagulants	16 (16.2)
Antidiabetics	6 (6.1)
Antidepressants	26 (26.3)
Calcium	16 (6.2)
Vitamin D	59 (59.6)

BMI, Body mass index; SLEDAI, Systemic Lupus Erythematosus Disease Activity Index; SLICC, Systemic lupus international collaborating clinics, VAS, Visual analogue scale; DMARDs, disease modifying anti-rheumatic drugs; M, mean; NSAIDs, nonsteroid anti-inflammatory drugs; SD, Standard deviation.

**Fig. 1:**
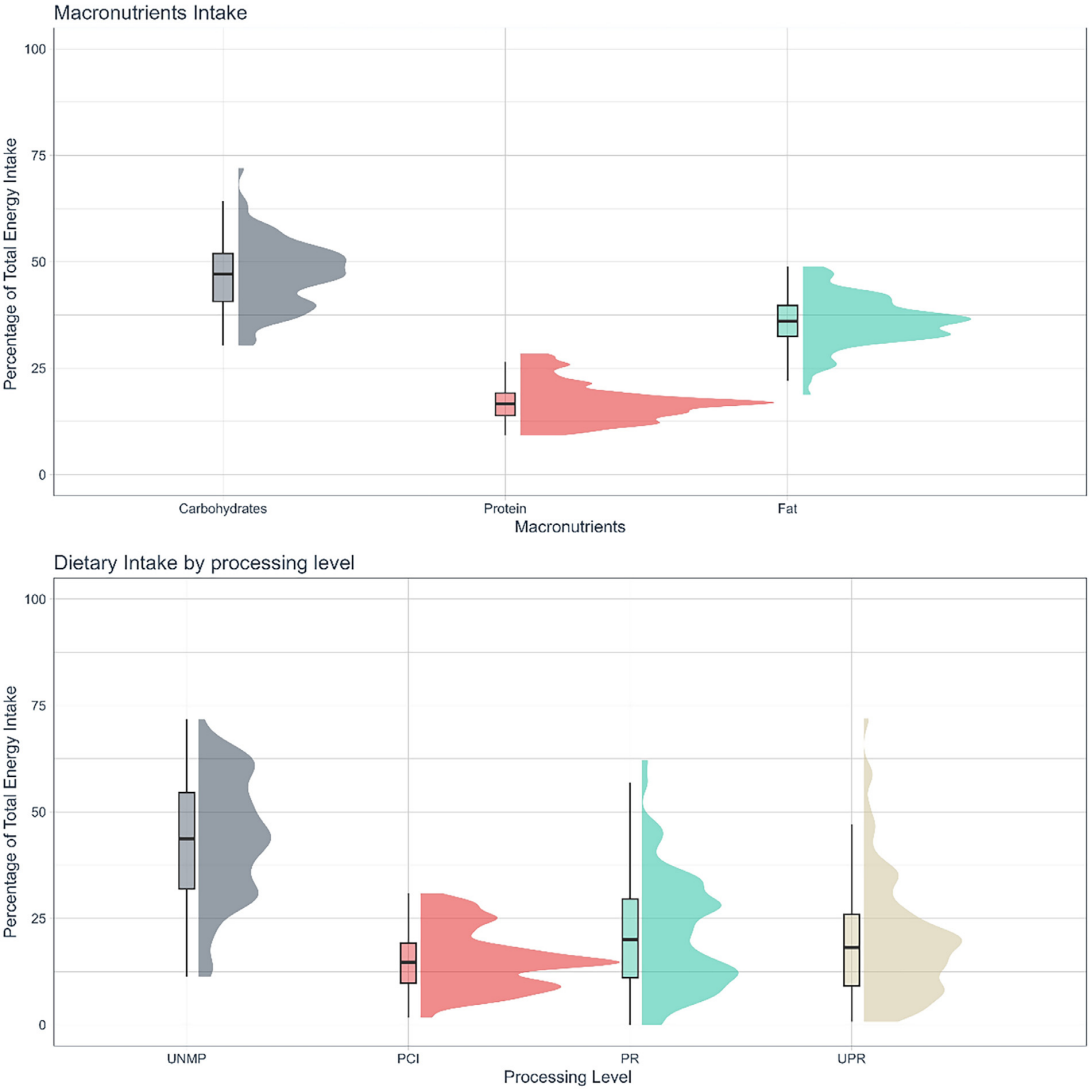
Food consumption. Relative energy intake (% kcal) by A) macronutrients and B) food processing level.

Average intakes were 47.1 ± 7.7%TEI, 17.0 ± 4.4%TEI, and 36.0 ± 5.7%TEI for carbohydrates, protein, and fat respectively (Fig. 1A, Table 2). Average fiber and calcium intake were below RDA: 16.1 ± 8.5g, 391.2 ± 217.8 mg, respectively (Table 2). Sodium intake was, on average, above recommendations: 2,919.0 ± 1,292.4 mg.^19^The most prevalent food group consumed was UNMP (43.8 ± 14.0% TEI), followed by PR (21.4 ± 13.1% TEI), UPR foods (20.0 ± 13.9% TEI), and PCI (15.4 ± 7.0% TEI) (Fig. 1B, Table 2).

**Table 2:** Food consumption, physical activity level, aerobic and functional capacity in systemic lupus erythematosus patients with high cardiovascular risk profile.

	M ± SD
**Energy (Kcal) (*n* = 92)**	1613.5 ± 649.2
**Macronutrients (*n* = 92)**	
Carbohydrate (TEI%)	47.1 ± 7.7
Fat (TEI%)	36.0 ± 5.7
Protein (TEI%)	17.0 ± 4.4
Protein (g/kg)	0.9 ± 0.5
**Micronutrients (*n* = 92)**	
Fiber (mg)	16.1 ± 8.5
Sodium (mg)	2919.0 ± 1292.4
Calcium (mg)	391.4 ± 217.8
**Food consumption by processing level (*n* = 92)**	
UNMP (TEI%)	43.8 ± 14.0
PCI (TEI%)	15.4 ± 7.0
PR (TEI%)	21.4 ± 13.1
UPR (TEI%)	20.0 ± 13.9
**Sedentary Behavior (hours/day) (*n* = 99)**	8.2 ± 2.2
**Physical activity level (*n* = 99)**	
Light physical activity (minutes/day)	83.2 ± 32.1
Moderate-to-vigorous physical activity (minutes/day)	13.0 ± 11.4
Moderate-to-vigorous physical activity (minutes/week)	91.2 ± 79.9
Number of steps (per day)	6782 ± 2720
**Aerobic capacity (*n* = 81)**	
Resting heart rate (beats/ minute)	70.5 ± 11.3
Peak heart rate (beats/ minute)	160.0 ± 19.4
VO2 anaerobic threshold (mL/kg/min)	11.8 ± 3.5
VO2 respiratory compensation (mL/kg/min)	19.1 ± 4.3
VO2peak (mL/kg/min)	21.2 ± 4.6
**Functional capacity (*n* = 90)**	
Timed stands (number of repetions)	11.2 ± 2.7
Timed up and go (seconds)	8.1 ± 2.3
Test Handgrip (kg/F)	26.4 ± 6.2

TEI, Total Energy Intake; UNMP, Unprocessed or Minimally Processed Foods; PCI, Processed Culinary Ingredients; PR, Processed Food; UPR, Ultraprocessed Food; Kg, Kilograms; F, Force; M, Mean; SD, Standard Deviation.

**Fig. 2:**
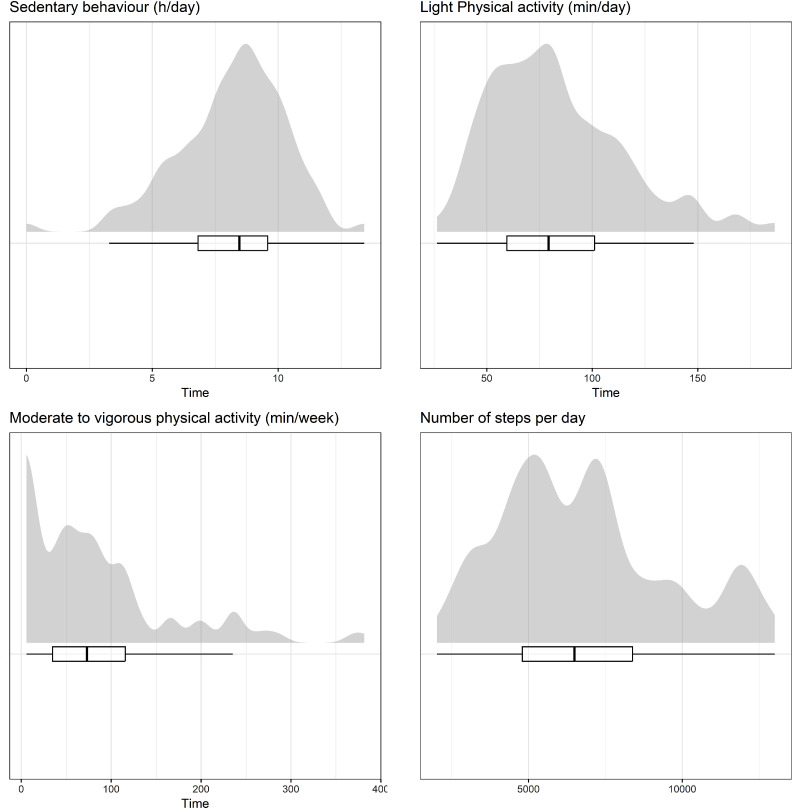
Physical activity level. Time spent in sedentary behavior, number of steps per day, level of light physical activity and moderate to vigorous physical activity.

None of the patients met recommendations for moderate to vigorous physical activity,^26^ and 58.6% of the patients spent more than 8 hours/day in sedentary behavior. Patients’ time spent in sedentary behavior was 8.2 ± 2.2 hours/day and the average light physical activity, moderate-to-vigorous physical activity, and number of steps were 83.2 ± 32.1 min/day, 13.0 ± 11.4 min/day (91.2 ± 79.9 min/week), 6782 ± 2720 (number), respectively (Fig. 2, Table 2).

The average results for Timed stands, Timed-Up-and-Go, and handgrip were 11.2 ± 2.7 repetitions, 8.1 ± 2.3 seconds, and 26.4 ± 6.2 kg/F, respectively. Average resting and peak heart rate were 70.5 ± 11.3 and 160.0 ± 19.4 beats per minute, respectively. Patients exhibited an average relative anaerobic threshold of 11.8 ± 3.46 (mL/kg/min), relative respiratory compensation point of 19.1 ± 4.30 (mL/kg/min), and VO2 peak of 21.2 ± 4.55 (mL/kg/min) (Table 2).

## Discussion

The present study aimed to characterize food consumption, physical activity level, sedentary behavior, and functional and aerobic capacity in a sample of SLE patients with high cardiovascular risk. Our findings show that: (i) several participants did not meet macronutrient and some of the micronutrients (e.g., fiber, calcium, and sodium) recommendations; (ii) the most prevalent food group (e.g., processing level) in our cohort's diet was UNMP, followed by PR, UPR and PCI; (iii) 58.6% of the patients exhibited high sedentary behavior (above 8h/day) and eighteen of them meet with minimum recommendation for moderate-to-vigorous physical activity; and (iv) functional and aerobic capacity (97.5%) on average were below that of the general population.

Increased CVD risk is a common feature in SLE.^3^ In fact, CVD is the main cause of morbimortality in these patients.^3^ Besides disease-related risk factors, poor lifestyle may prone patients with SLE to an even greater CVD risk.^12^^,^^14^^,^^15^ Namely, food consumption and physical activity are modifiable risk factors that may yield cardio-protective effects,^6^^,^^7^which may be especially important in patients with high CVD risk. In our sample, we found that although only a small part (4.3%) of the sample showed inadequate carbohydrate intake, a substantial part (35%) was below the protein recommendation, whereas 25% of the sample ingested more fat than the recommendation. Moreover, similar to previous observations in other populations with SLE,^27^we found insufficient fiber and calcium intakes in virtually every individual in our sample. In fact, while 81 patients did not meet fiber, none met adequate dietary recommendations for calcium intake. Additionally, only 24 participants did not exceed recommendations for sodium dietary intake. Adequate intake of fiber is responsible for better digestive health, glycemic control, and cholesterol regulation; calcium, in turn, is essential for bone health, blood coagulation and muscle and nerve functions. Sodium is an essential mineral in water balance, muscle and nerve functions and blood pressure control,^28^rendering proper monitoring of dietary intake in these patients essential to avoid worsening disease-associated comorbidities.^29^

Some studies have already demonstrated that higher consumption of UPR foods is associated with chronic non-communicable diseases, such as overweight and obesity, as well as diabetes, hypertension, and some types of cancer.^8^^,^^9^ The prevailing theoretical mechanism is associated with the fact that UPR foods contain high amounts of calories, sugar, fat, and salt, and low amounts of fibers, protein, vitamins, and minerals.^8^^,^^9^On the other hand, UNMP is rich in vitamins, minerals, and fibers, which may lead to anti-inflammatory and cardioprotective effects. Considering that SLE patients are at a greater risk of developing CVD (e.g., diabetes, hypertension, etc.)^3^ promoting a diet lower in UPR foods and, at the same time, higher in UNMP foods could improve cardiovascular risk profile in these patients.^20^

In the present study, the authors found a predominant consumption of UNMP foods, which is in line with previous observations in other rheumatic diseases,^30^ including a SLE cohort that reported a high consumption of UNMP and a low consumption of UPR foods.^29^ Indeed, the Brazilian population has a higher consumption of UNMP and lower consumption of UPR foods when compared to other countries (mainly developed ones).^31^The latest household budget survey (POF) carried out in Brazil between 2017‒2018 showed that UNMP foods are predominant while UPR is the least present food group. Nonetheless, our sample reported a slightly higher consumption of UPR than that of the general population (20.0% vs. 18.4% of TEI).

In addition to food consumption, the physical activity level, sedentary behavior, and functional and aerobic capacity were assessed in this study. The Brazilian guideline and the World Health Organization (WHO) recommend at least 150 to 300 minutes of moderate to vigorous aerobic activity per week for all adults and limit the amount of time in sedentary behavior.^26^^,^^32^ However, only eighteen of our patients reached the minimum recommended amount of moderate-to-vigorous physical activity. Indeed, our results are in agreement with previous data demonstrating that patients with rheumatic diseases spend a significant amount of time in sedentary behavior and have very low levels of physical activity.^11^^,^^12^ The low levels of physical activity observed in this population could be explained by socioeconomic, cultural and individual, and disease-related factors, such as time constraints, lack of motivation, financial costs, restricted access to specialized facilities, pain, fatigue, and concerns about worsening illness symptoms.^33^ Our sample also exhibited poorer aerobic and functional capacities compared to the general population,^34^ which is in line with previous literature.^35^ Reduced aerobic and functional capacity have been linked with difficulties in performing physical activities and simple daily tasks, such as climbing stairs, lifting heavy objects, or walking long distances.^36^^,^^37^In addition, considering the ACSM recommendations, most patients (97.5%) also had a lower than expected functional and aerobic capacity for women at the same age.

Strengths of this study are the homogeneous sample of patients with SLE who share a relatively unexplored characteristic, namely a high cardiovascular risk; and the use of objective measures to assess sedentary behavior, physical activity level, and functional and aerobic capacity. However, this study is not without limitations. Its cross-sectional design does not allow to establish causal relationships between variables, and the findings may not be widely generalized due to the specific inclusion criteria that were used in selecting our patients from a clinical trial. Additionally, there is an inherent bias associated with 24-hour food recalls (i.e., memory bias, underreporting of food consumption, lack of accuracy in recalling meals, and difficulties in estimating portion sizes). Nevertheless, participants were instructed to fulfill the 24-hour food recalls with maximum details regarding foods and drinks consumed, including portion sizes and recipes, and they also received the orientation to ask for help if necessary. Furthermore, researchers reviewed all 24-hour food recalls and when identified insufficient data, they tried to contact the participant to obtain the information. Finally, it is crucial to consider that our data was gathered during or after the most severe stage of the COVID-19 pandemic in Brazil, which may have affected physical activity and food consumption patterns.^38^, ^39^, ^40^

In conclusion, this study brings novel data on habitual food consumption and sedentary behavior, physical activity level, and functional and aerobic capacity in a sample of SLE patients with high cardiovascular risk. We found that while some aspects of dietary intake may be considered adequate (e.g., a high consumption of UNMP), others require attention in order to mitigate disease-related CVD risk (e.g., inadequate intake of macro and micronutrients). Overall, patients are physically inactive and spent a significant amount of time in sedentary behavior, which may partially contribute to their worsened aerobic and functional capacity, ultimately increasing their already risk for CVD. Data from this study may aid in the design of dedicated clinical trials aiming to investigate the effects of novel lifestyle interventions able to offset the risks of CVD in SLE.

## Data availability statement

The data that support the findings of this study are available from the corresponding author, B.G., upon reasonable request.

## Authors’ contributions

F.I.S., B.C.M., S.M.S., H.R. and B.G. participated in the conception and design of the study. F.I.S., B.C.M., S.M.S., MR, T.T.R. and M.C.A. are involved in patients’ recruitment and data collection. S.P., A.L.S.P. and F.R.L. are involved in patients’ screening and clinical assessments. F.I.S., B.C.M., S.M.S., M.R., F.B.B., H.R., and B.G. will be in charge of the statistical analyses, data interpretation, and manuscript writing. F.I.S., B.C.M., S.M.S., H.R. and B.G. drafted the manuscript with critical revision from S.P., A.L.S.P., F.R.L. and F.B.B. All authors have seen and approved the final version of the manuscript for publication.

## Conflicts of interest

The authors declare no conflicts of interest.
